# Supplementary material for: High-throughput detection of RNA processing in bacteria
Source: BMC Genomics. 2018 Mar 27;19:223. doi: 10.1186/s12864-018-4538-8 (PMC5870498; doi:10.1186/s12864-018-4538-8)
Supplement: Supplementary file 1 — Supplementary results, figures and methods. Figure S1. Measures of cleavage site motif category sequence member distance within and between categories. Figure S2A and B. Additional cleavage site motifs. Figure S3. While our data is more comprehensive, 86% of previously published transcription start sites lie within ±3 nt of our dRNA-Seq set. Figure S4. A Promoter motif associated with several virulence factors and two hypothetical proteins is highly similar to the RpoN binding motif. Figure S5. Small non-coding RNA gene expression in biofilms (A) and cells undergoing swarming motility (B) compared to expression in planktonic cells. Figure S6. Proposed polymerase dimer binding to a palindromic antiparallel TSS. Figure S7. Step by step construction of the pRNA-Seq library. Figure S8. Peak height threshold determination for generating cutoffs for site predictions (Transcription start sites (TSS) and RNA cleavage sites). Table S1A. SNPs found in our sequenced isolate of PAO1 cf. the reference strain. Table S1B. Indels found in our sequenced isolate of PAO1 vs. the reference strain. Table S2. The 240 most abundant transcripts from the RNA-Seq study of cells grown in LB at 37 °C. Table S3. Sequence motifs associated with cleavage sites. Table S4. p-values for promoters matching motif associated with virulence factors.Table S5A. Summary of predicted RpoN binding sites and adjacent downstream genes in Pseudomonas aeruginosa PAO1. Table S5B. Function classes associated with genes downstream of predicted RpoN binding sites. Table S6. Fold change in RNA-Seq read density throughout the PAO1 genome. Table S7. Sequences of the oligonucleotides used in the preparation of the pRNA-Seq library. Table S8. Fitted slopes and intercepts for data from H = 1..100XX. Fit to G(H) = H0XS. Table S9. List of primers utilized for confirmation of sRNAs by RT-qPCR. The Perl script used for 5′ end trimming on library. (DOCX 1536 kb) [file 12864_2018_4538_MOESM1_ESM.docx]

**SUPPLEMENTARY INFORMATION**

**RESULTS**

**RNA cleavage patterns correlated with RNA cleavage motifs**

The next two most frequent RNA cleavage patterns were defined by sites where two adjacent RNA cleavage events occurred with high probability, the less abundant of the two being either 5’ (45/383), “Twin L”, [(A,c,g,u)(A,C,u)(G,a,c,u)(G,a,c,u) ↓ (A,c,u)(C,U)(A,G,c)(a,c,g,u)(G,a,c,u)] (Fig. S1A), or 3’ (33/383), “Twin R”, [(A,c,g,u)(C,a,g,u)(G,a,c,u)(A,c,g,u) ↓ (A,c,u)(C,U)(**A**)(C,U,a)(A,c,g)] (Fig. S1B). These indicated a “tail” or “shoulder” pattern, depending on the abundance of the secondary cleavage event relative to the primary one. When the 3’ site was the most prominent, as might occur due to a nearly completely processive 5’->3’ endonuclease digestion, distinct sequence motifs were again observed. RNAs containing Twin R cleavage sites were most prominently transcribed from genes associated with oxidative phosphorylation (number of genes = 5, corrected p-value = 0.00097) and protein export (number of genes = 3, corrected p-value = 0.0059). Genes from which Twin L transcripts were produced were not significantly associated with any KEGG categories. In addition to the above, there were 11 cleavage sites that did not fit into any of the above categories, and for which no distinct cleavage pattern was observed. Figure S1 shows a Euclidian distance analysis that was conducted to determine how different the five sequence motif categories are from each other and within each category.

**The utility of promoter data: Approach I – Identification of novel virulence regulon members.**

A search for genes sharing promoter motifs similar to those of virulence factors was initiated, with the aim of identifying hypothetical proteins that might be regulated by the same transcription factor and that may also play a role in the virulence processes. We calculated similarities between promoter motifs as pairwise distances using MEGA v.5.05 with default parameters for all promoters [71]. A list of all known virulence factors in *P. aeruginosa* PAO1 was obtained from the *Pseudomonas* Genome Database [16]. The promoter for one virulence factor, the flagella biosynthesis protein PA1452/flhA, which plays a role in adherence and biofilm development [72], and the 10 most similar promoters to the virulence factor promoter were used for motif prediction using MEME v4.8.1 [68] with default parameters (see Fig. 7 for a depiction of the motif and Table S4 for a list of the genes downstream of the promoter motif). Of the 10 promoters recovered in this search, three were upstream of genes involved in the biogenesis of the virulence factor flagellin and three were upstream of hypothetical proteins. The promoter motif consensus for the genes listed in Table S4 closely matched (e-value = 4.8 x 10^-21^) that for genes regulated by the non-canonical Sigma factor RpoN (see Figure S4), as assessed by TOMTOM v4.8.1 [73]. The promoter region of PA4739 contained regions that were highly similar to the RpoN -12 and -24 motifs. This gene encodes a conserved hypothetical protein that is predicted to localize to the periplasm [74]. PA4739 is upregulated when PAO1 cells are exposed to human respiratory epithelia [75], is downregulated by treatment of biofilm cells with the anti-biofilm peptides 1037 and LL-37, and when mutated leads to significantly reduced biofilm production by *P. aeruginosa* [76]. This analysis illustrates how genes such as PA4739 can be identified as candidates for further investigation for a possible role in virulence, or other functions potentially co-regulated under certain conditions with known virulence factors.

**The utility of promoter data: Approach II – Sigma factor binding site identification**

We also investigated whether we could discover putative binding sites for known sigma factors in our datasets. We chose RpoN (Sigma54) as a case study due to its well-annotated binding sites including experimentally verified locations that are defined in the PRODORIC database [15]. Six sequences matching the core RpoN-binding motif [10,77] were aligned using ClustalW [80], with default parameters, while the Emboss (v. 6.2.0) program Prophecy [78] was used to generate a Gribskov position-specific weighted profile from this alignment. The Gribskov profile was used to search for promoter regions upstream of our predicted TSSs. A total of 32 putative RpoN binding sites matching the profile with 85% or better confidence were identified in the upstream promoter regions. Three of these genes were also predicted to have RpoN binding sites in the pairwise distance and motif analysis of promoters described above. Of the 32 binding sites identified here, 25 were upstream of genes not previously described to be regulated by RpoN, while 4 were upstream of genes previously known to be regulated by this Sigma factor, but for which RpoN sites had not been described (Table S5A). While 6 of the genes, putatively regulated by RpoN have no known function, several were associated with functions already attributed to RpoN-regulated genes (Table S5B), including motility and attachment, adaptation/protection and transport of small molecules [80–82]. The promoter for PA4739 was also recovered through this search method (96% confidence match to the RpoN motif), implying that it might be directly regulated by RpoN. Three of the six sequences used as input for constructing the original Gribskov profile were also identified in the set. The other three motifs were missing from the set because their associated promoter regions lacked TSS predictions (likely due to lack of expression under the analyzed conditions).

**Confirmation and identification of ncRNAs**

Most of the 26 ncRNAs were conserved in all 6 sequenced strains of *P. aeruginosa*, with two (3930282-3930639 and 3545572-3545872) being restricted to strain PAO1 (data not shown). Three of these ncRNAs were not previously identified, with Gomez-Lozano et al. [35] failing to identify 4 of the 26 novel transcripts and Wurtzel et al. [17] not observing 15 (two of which had no homologs in strain PA14 in which they performed their studies). Such ncRNA species have been proposed to be involved in a variety of regulatory functions, often acting transcriptionally or post transcriptionally. To highlight this feature, we performed RT-qPCR on each of the 31 ncRNAs (26 novel and 5 previously identified) under planktonic conditions (i.e. free swimming in medium) as well as biofilm formation and swarming motility, representing complex adaptations (lifestyle changes) whereby *Pseudomonas* undergoes far-ranging physiological, regulatory and gene expression changes. Our studies demonstrated that 30 of the 31 ncRNAs verified through RT-qPCR were modestly to considerably differentially regulated (Table 3, Figure S5) under one or both conditions of biofilm growth or swarming motility. RsmY and PrrF1-2 (PrrH), served as controls, since they had been previously shown to be up-regulated during the biofilm mode of growth [24]. There was no absolute pattern observed since overall we observed coordinate, inverse or unique regulation of individual ncRNAs during these complex adaptations, and both up and down regulation were observed. We hypothesized that ncRNAs are intimately associated in tailoring the lifestyle changes associated with biofilm formation and swarming motility. Consistent with this, previous studies have implicated ncRNA species *crcZ* [24,49] and *rsmXY* [24,52,85] in biofilm formation or swarming motility, while our own preliminary studies showed that *prrF1-2* and *phrS* regulates swarming motility and to a lesser extent biofilm formation (data not shown).

**METHODS**

**Cutoff identification: Peak Height Threshold Determination for TSS and RNA cleavage site predictions**

Cutoffs/thresholds for predicting TSS and RNA cleavage sites were determined logically. By analyzing the number of TSS peaks of a given height, G(H), as a function of the peak height (H) peak height thresholds were identified, for both the dRNA-Seq and pRNA-Seq data sets, to enable the identification of peaks highly likely to be derived from a biological process. For low H, a log-log plot of G(H) versus H fitted the equation G(H) = H_i_(H)^S^ for both the dRNA-Seq and pRNA-Seq data (Fig. S8 and Table S8). Interestingly the dRNA-Seq library had large H_i_ intercept values that were consistent with the size of the *P. aeruginosa* PAO1 transcriptome (~1/10 the genome size having H_i_ values very similar to that were observed in the RNA-Seq libraries: Table S8). Both the plus and minus strand data had very similar H_i_ and S values in the dRNA-Seq libraries suggesting that the sequencing methodology was responsible for the observed power law distribution of low H peaks. The plus and minus dRNA-Seq G(H) curves both had a shoulder at an H value around 100. Beyond this point the slope of G(H) changed abruptly and G(H) simultaneously became more dispersed. It is this region (where H > 100) of the dRNA-Seq data that had high biological significance, with expression on the plus and minus strand having clearly distinct profiles (Fig. S8, where plus and minus strand transcripts are shown as blue and red respectively). For the dRNA-Seq analysis we set a conservative threshold of H(dRNA)_cut_off_ = 500 to robustly identify, from a random mapping process, the most significant peaks/coverage .

In contrast, the plus and minus strand data for the pRNA-Seq libraries were significantly different from one another. This appears to be related to the differences observed in the high peak height values for the dRNA-Seq dataset. At low H, H_i_ was significantly smaller than found for the dRNA-Seq data (Fig. S8). The minus strand had a significantly higher H_i_ value than the plus strand. This finding would not be expected due to a random non-biological process that would be expected to be strand-independent and may correspond to the fact that considerably more rRNA is encoded on the minus strand than the plus [1]. Interestingly, the H_i_ intercepts for the low H pRNA-Seq data corresponded well to intercepts drawn for the high H dRNA-Seq data. One interpretation of this data is that random cleavage of RNA transcripts is responsible for the low H_i_ intercepts. While the random degradation of RNA encoded in the monoP library would be of potential interest, random cleavage events were excluded by setting an H(pRNA)_cut_off_ = 100. This is due to the complexity of the data and the difficulty of discerning completely random events from those with biological consequence where only a small amount of read data is available. While the pRNA-Seq and dRNA-Seq cutoffs were used as a basis for site predictions (see below), note that the original read coverage data and predictions, are also available in the Jbrowse view of the data at [www.pseudomonas.com](http://www.pseudomonas.com).

**Novel regulon member identification**

To analyze whether motifs present in the promoters of genes belonging to a particular functional class could be used to identify genes belonging to the same class that had been previously classified as hypothetical or unknown, we utilized genes encoding virulence factors as a test case. Pairwise distances were calculated for all promoter sequences (regions spanning nucleotides -4 to -40 bp upstream of the TSS) for 1599 TSS using MEGA v5.05 [71] with default parameters. The list of all known virulence factors in strain PAO1 was obtained from the *Pseudomonas* Genome Database [16]. A virulence factor of interest (flhA, PA1452) and its 10 most similar sequences were used for motif prediction using MEME v4.8.1 [68] with default parameters. Additionally, TOMTOM v4.8.1 [73] was used to compare the MEME predicted motif to the motif curated in the PRODORIC Database (Release 8.9) [15].

**RpoN Binding Site Identification**

To gain more insight into putative RpoN (sigma54) binding sites in PAO1, the PRODORIC website (http://prodoric.tu-bs.de/) [15] was utilized as a source of known binding sites. The 5 available sequences were aligned using ClustalW [71], with default parameters, and analyzed using Prophecy from the Emboss Software Package (v. 6.2.0) program [79] to create a Gribskov profile. A FASTA file was then created that contained the -41 to -4 regions upstream of all predicted TSS (based on PPP/dRNA-Seq data) where only the ‘primary’ start sites located upstream of genes were utilized. The program Emboss Profit was used to search this FASTA file and any hits with a threshold reporting percentage ≥ 85% were retained.

**Ribosomal Binding Site Prediction**

The PRODIGAL program for prokaryotic gene finding [31] utilizes ribosomal binding site (RBS) motifs as part of its iterative start training for identification of translation initiation sites. PAO1 gene predictions and corresponding RBS predictions and coordinates of their sequences were downloaded (http://prodigal.ornl.gov/downloads.php). This information was used to create a tally of how many cleavage events occurred in the following regions: 1) between TSS and RBS 2) within the RBS and 3) between RBS and translation initiation site.

**Small ncRNA identification**

Intergenic regions in *P. aeruginosa* PAO1 that were not annotated as belonging to an operon (according to PRODORIC [15]) were included in the sRNA identification process. Due to the fact that the reads from the standard RNA-Seq libraries are not strand-specific, only intergenic regions that were not within operons were examined. Intergenic regions of at least 200bp in length and containing a median number of RNA-Seq reads (in library PA0004) per base over the length of the region ≥100 were subjected to BLASTX analysis. Only regions that had no BLASTX hits <10^-4^ in the nr database were examined manually for evidence of a ncRNA. ncRNAs were sought in all 4 RNA-Seq libraries, and compared to lists of known sRNAs and the Rfam database [48]. ncRNAs were required to have 50 reads in at least two of the four RNA-Seq libraries. At the time that searches for ncRNAs were initiated, the aim was identify novel transcripts. Many small ncRNA genes were complementary to other loci in the PAO1 genome. Complementarity was determined using the entire small ncRNA transcript sequences and the discontiguous megablast BLASTn suite (http://blast.ncbi.nlm.nih.gov) and searches were limited to *P. aeruginosa* sequences (taxid:136841).

**Functional analysis of small ncRNA by RT-qPCR**

Swarming motility in PAO1 was carried out as in previous studies [56]. Swarming colonies at the ends of tendrils were collected for RNA isolation. Biofilm growth was assayed by growing PAO1 statically at room temperature for 4 days and biofilms were collected from the air-liquid interface [57]. Whole cell RNA from mid-logarithmic growth, swarming and biofilm cultures was purified according to the protocol from the Qiagen RNeasy kit (cat.no.74104). For real-time, semi-quantitative PCR (RT-qPCR) using the primers laid out in Table S9. Differential expression of a given ncRNA transcript was considered to be significant if the average of three independent biological samples gave greater than 2-fold changes under respective swarming or biofilm conditions when normalized to planktonic mid-logarithmic growth PAO1.

**Figure S1: Measures of cleavage site motif category sequence member distance within and between categories.** The major diagonal set of boxes measures "within-ness", which is the sum of the Euclidean distance of the members of the same clusters to the center of the cluster. Off-diagonal boxes measure "without-ness", which is the sum of the euclidean distance of the members of the cluster i to the members of cluster j. In the heat map, dark colors indicate a shorter distance, and all values are in
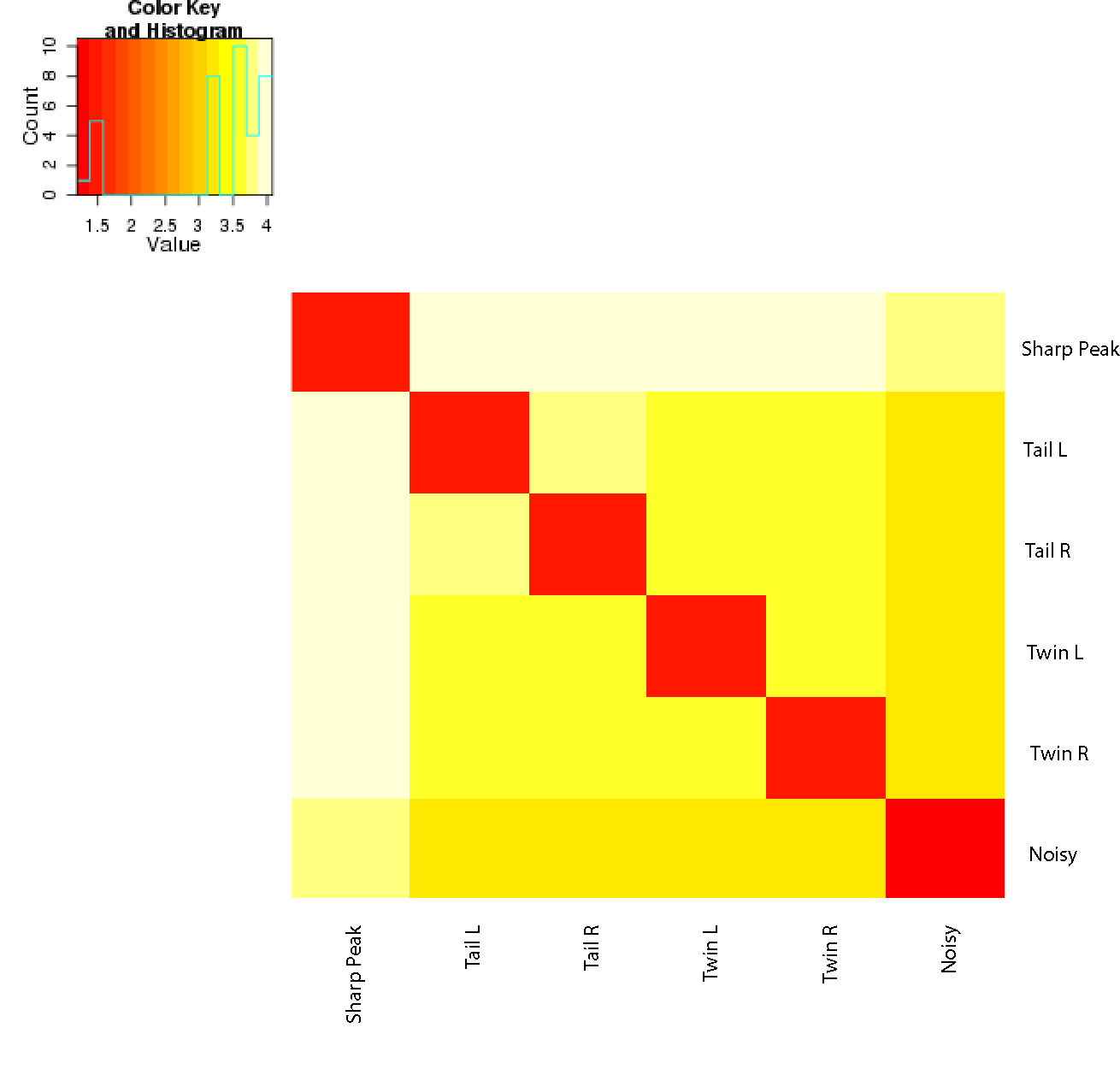
log10 scale.

**Figure S2: Additional cleavage site motifs.** Cleavage sites are genome locations with >=100 reads first bp coverage from the pRNA-Seq library. Peak shape refers to the number of mapped transcripts surrounding a cleavage site. Peak shapes were categorized using k-mean clustering. Motifs were calculated from peak shape clusters using MEME [68]. The graph at the top of each panel shows normalized peak height. The WebLogo [69] at the bottom of each panel shows the sequence motif associated with each peak shape. Panel A depicts the “Twinpeaks L” motif and panel B depicts the “Twinpeaks R” motif.


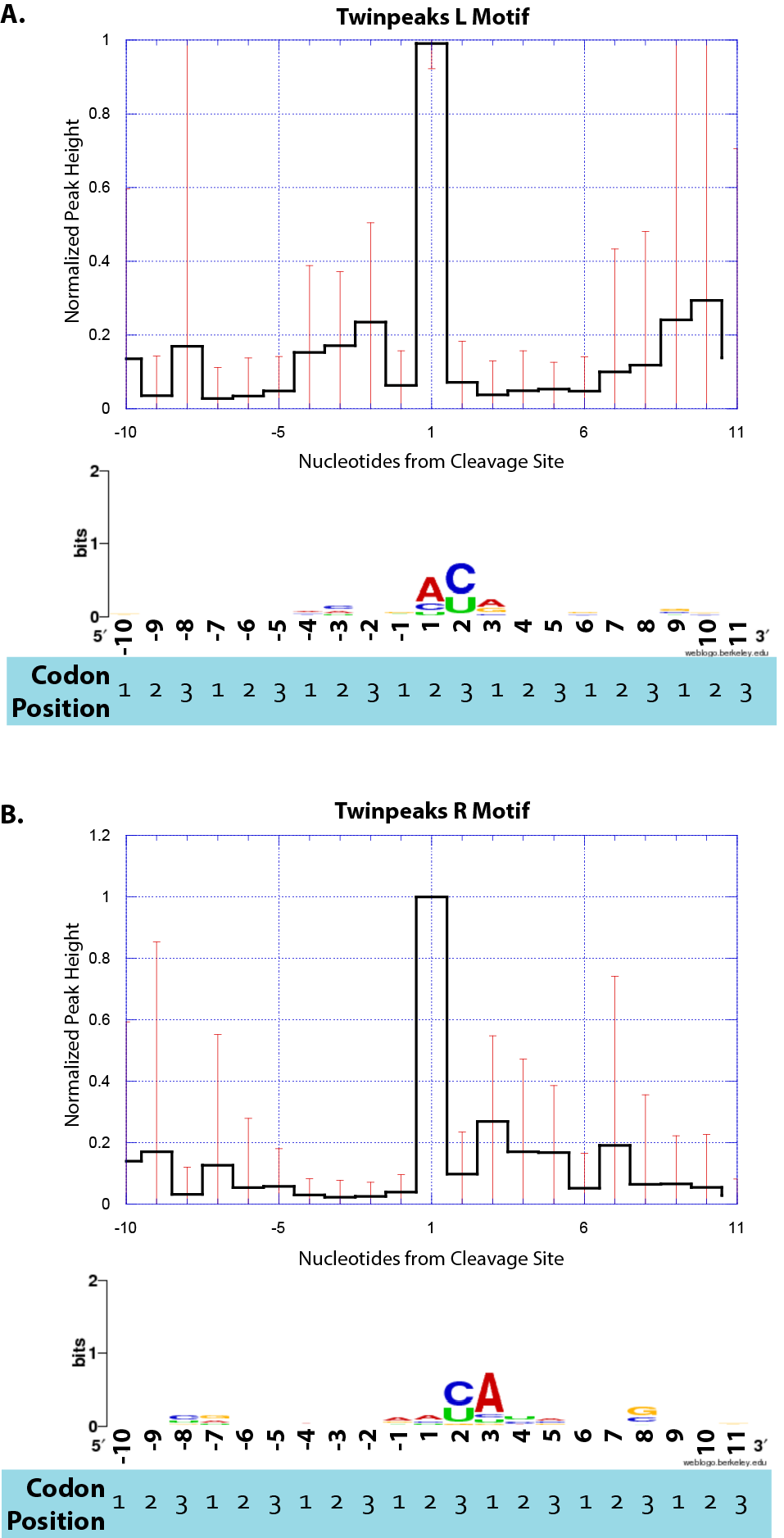


**Figure S3: While our data is more comprehensive, 86% of previously published transcription start sites lie within ± 3 nt of our dRNA-Seq set.** The difference between previously published TSS and our TSS was calculated by subtracting the genome coordinate of our TSS from the published TSS. Not all TSS data stored in PRODORIC were obtained from strain PAO1 grown in LB media (as done here for the dRNA-Seq library), and the data was mostly obtained from primer extension and S1 nuclease analysis experiments [84–86]. Primer extension and S1 nuclease analysis are capable of identifying promoters of varying strengths, while we imposed a strict read threshold of 500 (see methods for rationale) which might have caused us to overlook some weaker TSS. Therefore, in order to make our data comparable to that in PRODORIC, we excluded any TSS from our analysis that had been obtained in media other than LB, and lowered the 500 read threshold to 100 reads. We removed two TSS from our dataset, and found five TSS closer to annotated TSS using the 100 read threshold. 71% of TSS lie within ± 1 nt of previously annotated TSS, 78% lie within ± 2, and 86% lie within ± 3 nt. The difference between the two TSS is plotted on the X-axis in nucleotides (area shaded in blue).


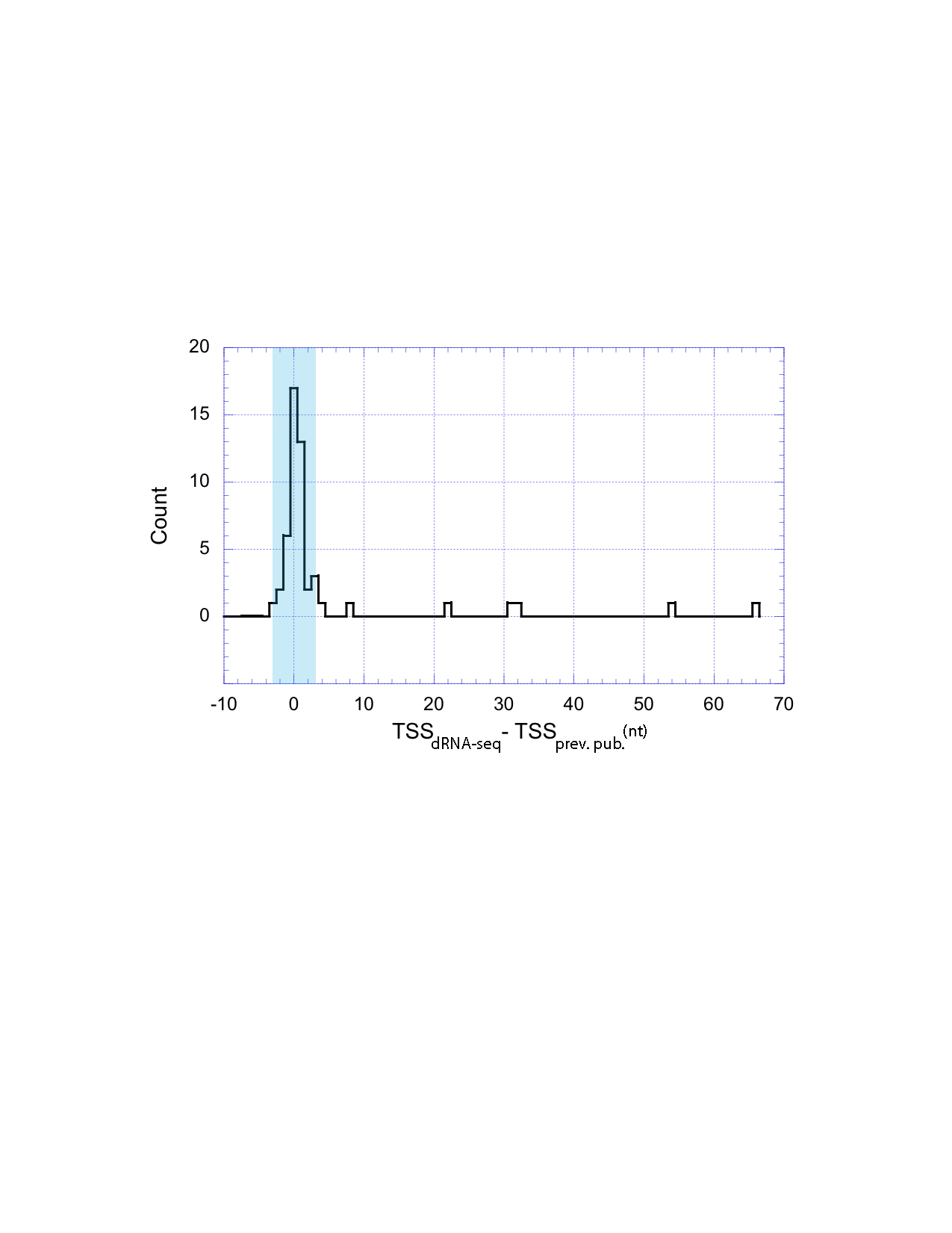


**Figure S4: A Promoter motif associated with several virulence factors and two hypothetical proteins is highly similar to the RpoN binding motif.** Pairwise distances between all promoter sequences were calculated using MEGA [71]. The virulence factor PA1452 was chosen as a query, and the 10 promoters with the closest pairwise distances were selected. MEME [68] was used to search for motifs within the promoters. Panel (A) shows the MEME motif. The two boxes with dashed lines enclose regions that are highly similar to the -12 and -24 regions of the rpoN binding site motif found in γ-proteobacteria, as shown in panel (B). The promoters highlighted in blue lie upstream of known virulence factors. The promoter highlighted in red lies upstream of PA4739, a hypothetical protein that is a prime target for further study as a gene possibly involved in virulence.


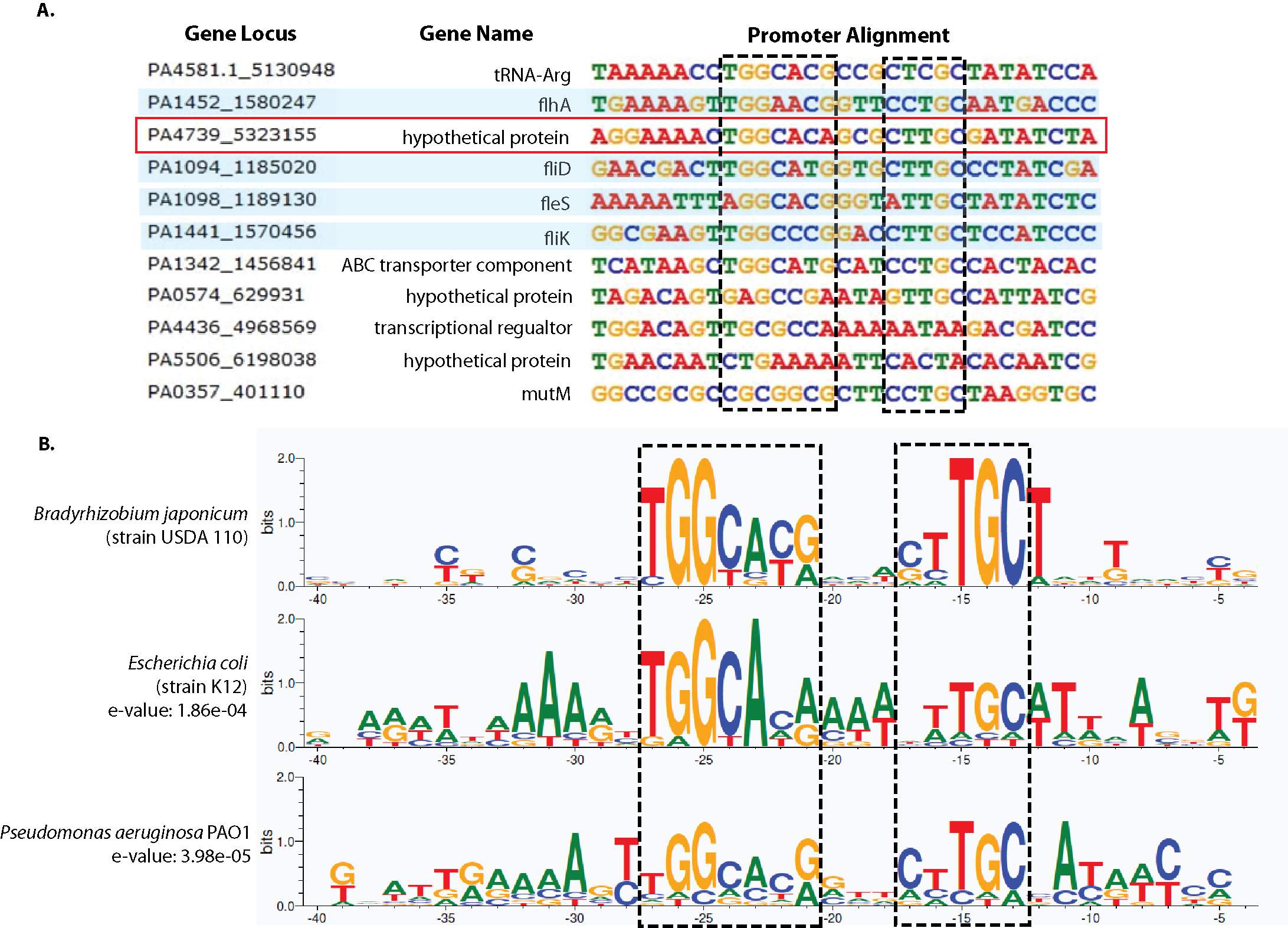


**Figure S5: Small non-coding RNA gene expression in biofilms (A) and cells undergoing swarming motility (B) compared to expression in planktonic cells.** Gene expression was determined by RT-qPCR and represents the mean of 3 separate biological repeats. Genomic coordinates are as presented in Table 3. RsmY and PrrF1-2, included as controls, were previously known to be up-regulated during the biofilm mode of growth [24].
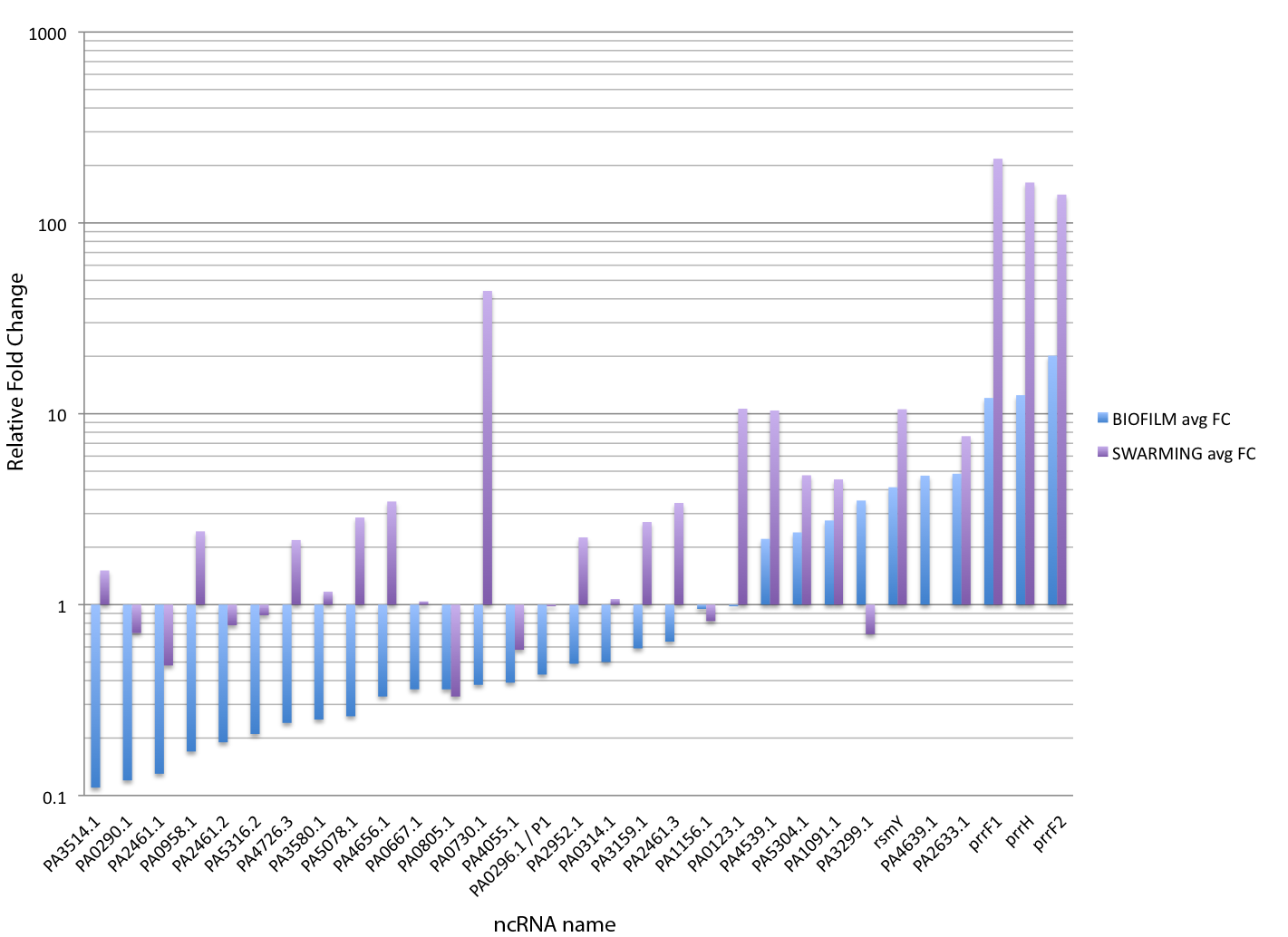


**Figure S6: Proposed polymerase dimer binding to a palindromic antiparallel TSS.** A polymerase dimer is shown bound to a region containing antiparallel transcription start sites and palindromic -10 elements, where transcription could take place in a competitive manner from either start site. The polymerase active sites are depicted as shaded gray circles. Within the region where the polymerase dimer is bound, only every third base is shown for simplicity. The two transcription start sites are 18 bases away from each other.

**
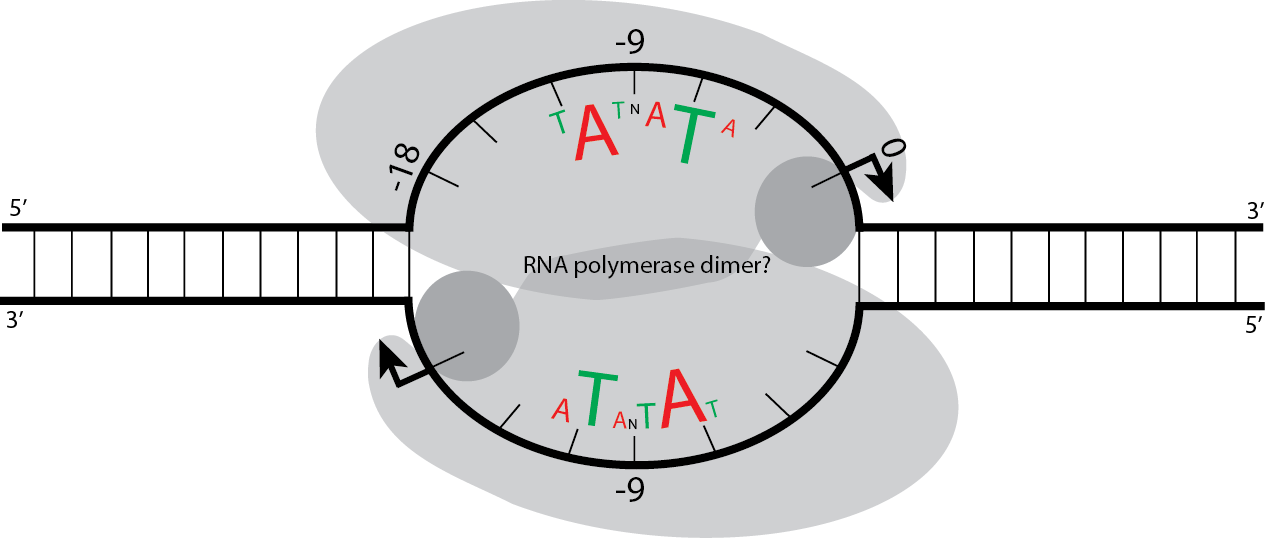
**

**Figure S7: Step by step construction of the pRNA-Seq library.** RNA = pink, cDNA = purple, DNA oligos = blue, and RNA oligos = green. Total cellular RNA is composed of three distinct species that have 5’ triphosphates (5’PPP), 5’ monophosphates (5’P) and 5’ hydroxyls (5’OH), respectively (A). Refer to Figure 1 of the main manuscript for an explanation of how these were generated. After treatment with DNase (not shown), the RNA was incubated with a DNA oligonucleotide (DNA 17.71) and T4 RNA ligase. All three species of RNA molecule possessed the same OH group on the 3’ end, therefore the RNA ligase would attach the DNA oligonucleotide to all types of RNA present (B). Incubation of the RNA/DNA hybrids with RNA ligase and an RNA oligo would only allow ligation of the RNA oligo (RNA 17.50) onto the 5’ end of RNA/DNA hybrids that have 5’ monophosphates (C). The next step was reverse transcription of the DNA/RNA hybrids with a primer that was complimentary to DNA 17.71 (DNA 16.16) (D). PCR amplification only amplified cDNA molecules that had both the DNA and RNA oligos ligated onto the ends of the original RNA, or RNA that initially contained a monophosphate at the 5’ end (E). Therefore, a pRNA-Seq library would only be generated from RNAs that initially had a monophosphate at the 5’ terminus.

**
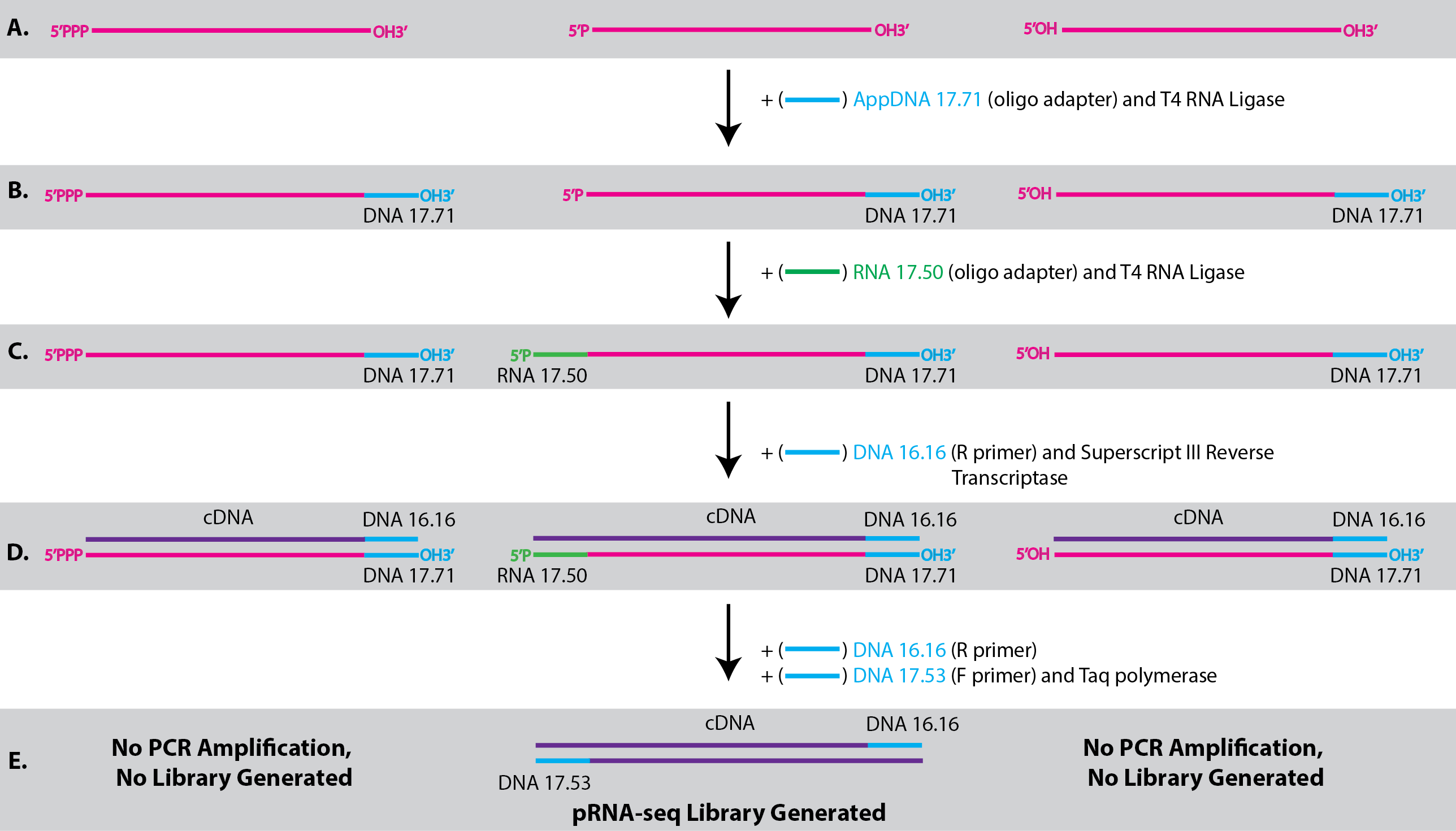
Figure S8: Peak height threshold determination for generating cutoffs for site predictions (Transcription start sites (TSS) and RNA cleavage sites).** Peak height (H) was calculated at each genomic locus, the number of such peaks across the genome G(H) was then calculated. Linear curves observed on the log-log plot for H less than about 100 likely indicate random events due to sequencing (for the TSS library the y-axis intercept is approximately the transcriptional coverage in bp under this assumption). For H larger than about 100 there is a distinct change in slope suggestive of a biological origin. Color key: Blue dRNA-Seq (TSS) +strand, red dRNA-Seq (TSS) –strand, black pRNA-Seq +strand, green pRNA-Seq –strand.


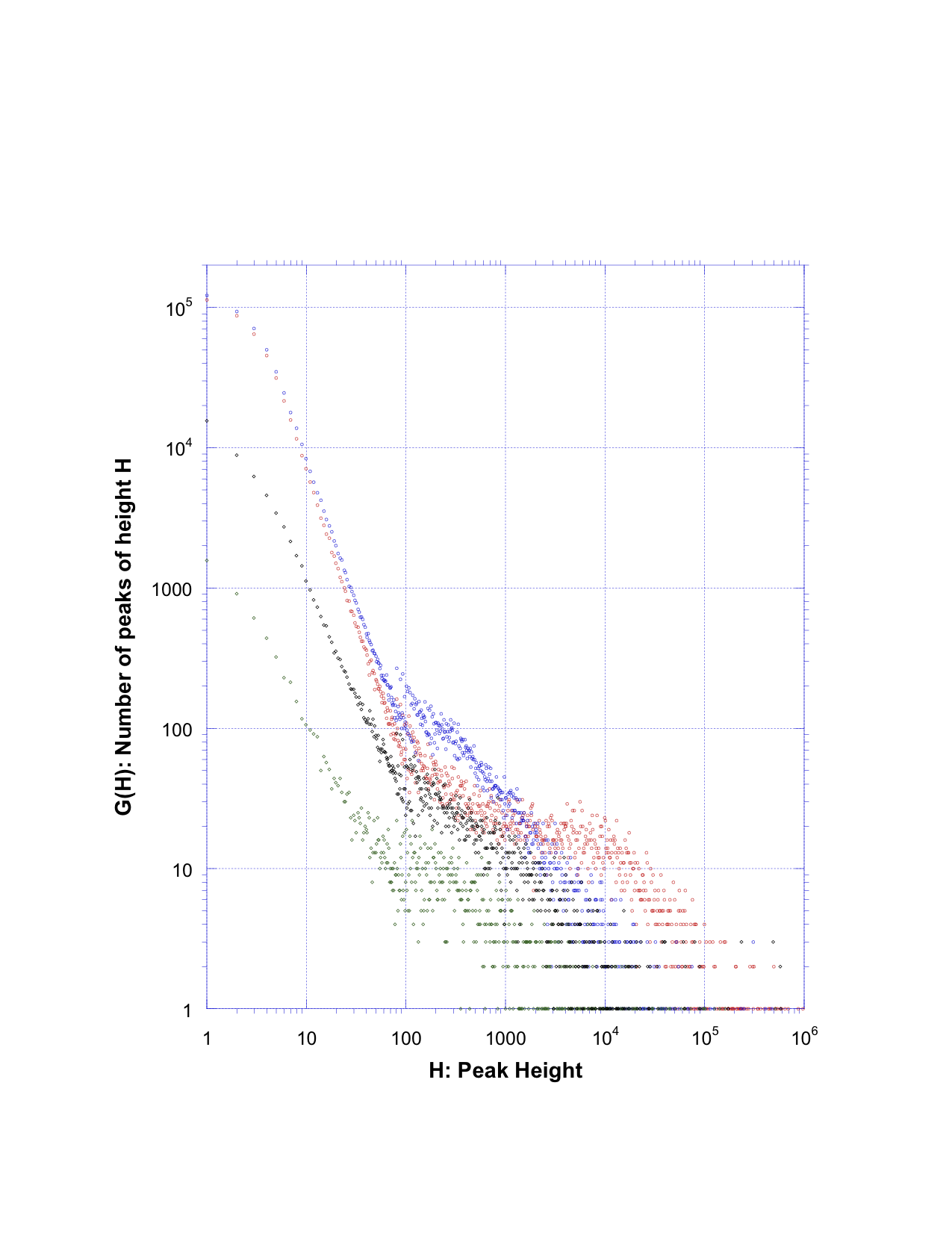
**Table S1A:** **SNPs found in our sequenced isolate of PAO1 cf. the reference strain.** SNPs in our PAO1 genomic library were determined using Bowtie [59], BWA [60], mrsFAST [61] and SSAHA2 [62] software. The SNPs shown in the table below were detected using all of four of the algorithms. Each SNP’s position in the genome, locus, protein, nucleotide change and amino acid change are shown where applicable.

| **Position in Genome** | **Locus** | **Protein** | **Nucleotide Change** | **Amino Acid Change** |
| --- | --- | --- | --- | --- |
| 169284 | Intergenic (PA0148-PA0149) | Not applicable | G -> C | n/a |
| 413850 | Intergenic (PA0369-PA0370) | Not applicable | T -> C | n/a |
| 1589438 | PA1459 | Chemotaxis-specific methylesterase | G -> C | G -> A |
| 2669175 | PA2400 | Probable nonribosomal peptide synthetase PvdJ | G -> C | P -> A |
| 4344266 | PA3877 | Nitrite extrusion protein 1 NarK | A -> G | Syn. |
| 4924552 | PA4394 | Hypothetical protein | C -> G | V -> L |
| 4924553 | PA4394 | Hypothetical protein | G -> C | Syn. |
| 5743461 | PA5100 | Urocanatehydratase HutU | C -> G | T -> S |
| 5743462 | PA5100 | Urocanatehydratase HutU | G -> C | T -> S |
| 6098781 | PA5418 | Sarcosine oxidase subunit SoxA | G -> C | Syn. |
| 6115455 | PA5434 | Tryptophan permease (Mtr) | T -> G | K -> N |

**Table S1B:** **Indels found in our sequenced isolate of PAO1 vs. the reference strain.** Indels in our PAO1 genomic library were determined using BWA [60] and SSAHA2 [62]. All indels listed in the table below were predicted using both algorithms. Each indel’s position in the genome, locus, protein and nucleotide are shown.

| **Position in Genome** | **Locus** | **Protein** | **Nucleotide** |
| --- | --- | --- | --- |
| 740419 | PA0683 | Prob. type II secretion system protein | G -> GC (insertion) |
| 1116213 | PA1029 | hypothetical protein | G -> GC (insertion) |
| 1445357 | Intergenic (PA1332-PA1333) |  | A -> AG (insertion) |
| 1835045 | PA1685 | Enolase-phosphatase MtnC | G -> GC (insertion) |
| 2239555 | Intergenic (PA2046-PA2047) |  | A -> AG (insertion) |
| 2355771 | PA2139 | hypothetical protein | A -> AG (insertion) |
| 2532046 | Intergenic (PA2300-PA2301) |  | G -> GC (insertion) |
| 2753522 | PA2452 | hypothetical protein | G -> GC (insertion) |
| 3083196 | PA2727 | hypothetical protein | A -> AG (insertion) |
| 4888194 | PA4360 | hypothetical protein | A -> AG (insertion) |

**Table S2: The 240 most abundant transcripts from the RNA-Seq study of cells grown in LB at 37^0^C.** Bolded rows indicate genes whose transcripts were also identified in the 5’ monophosphate cleavage study.

| **Start** | **End** | **Gene ID; name** | **Function** | **Number of Reads** |
| --- | --- | --- | --- | --- |
| **483** | **2027** | **PA0001; dnaA** | **Chromosomal replication initiator protein** | **30591** |
| **2056** | **3159** | **PA0002; dnaN** | **DNA polymerase III, beta chain** | **33226** |
| **4275** | **6695** | **PA0004; gyrB** | **DNA gyrase subunit B** | **63600** |
| 10434 | 12488 | PA0008; glyS | Glycyl-tRNA synthetase beta chain | 25791 |
| 101778 | 103274 | PA0084; tssC1 | Predicted component of the type VI protein secretion system | 30120 |
| 299522 | 300973 | PA0265; gabD | Succinate-semialdehyde dehydrogenase | 37556 |
| 301218 | 302498 | PA0266; gabT | 4-aminobutyrate aminotransferase | 44367 |
| 327284 | 328666 | PA0291; oprE | Anaerobically-induced outer membrane porin | 43059 |
| 336951 | 338321 | PA0299; spuC | Putrescine aminotransferase | 26905 |
| 338437 | 339540 | PA0300; spuD | Polyamine transport protein | 22961 |
| 355248 | 356477 | PA0316; serA | D-3-phosphoglycerate dehydrogenase | 25510 |
| 454126 | 461544 | PA0413; chpA | Component of chemotactic signal transduction system | 33719 |
| 473191 | 476331 | PA0426; mexB | Resistance-Nodulation-Cell Division (RND) multidrug efflux transporter | 63088 |
| 476333 | 477790 | PA0427; oprM | Major intrinsic multiple antibiotic resistance efflux outer membrane protein | 27673 |
| 477886 | 479805 | PA0428; | Probable ATP-dependent RNA helicase | 33392 |
| 482706 | 484115 | PA0432; sahH | S-adenosyl-L-homocysteine hydrolase | 39222 |
| 603706 | 604896 | PA0546; metK | Methionine adenosyltransferase | 39590 |
| 606160 | 608157 | PA0548; tktA | Transketolase | 39537 |
| 613338 | 614402 | PA0555; fda | Fructose-1,6-bisphosphate aldolase | 25909 |
| **634371** | **636224** | **PA0576; rpoD** | **Sigma factor** | **61509** |
| **652480** | **653772** | **PA0594; surA** | **Peptidyl-prolyl cis-trans isomerase** | **28690** |
| **653753** | **656527** | **PA0595; ostA** | **Organic solvent tolerance protein** | **65177** |
| 833949 | 835373 | PA0766; mucD | Serine protease MucD precursor | 24279 |
| **835523** | **837322** | **PA0767; lepA** | **GTP-binding protein** | **26234** |
| 851783 | 854965 | PA0782; putA | Proline dehydrogenase puta | 148652 |
| 855277 | 856797 | PA0783; putP | Sodium/proline symporter putp | 44042 |
| 864095 | 865510 | PA0789; | Probable amino acid permease | 33072 |
| 945834 | 946907 | PA0865; hpd | 4-hydroxyphenylpyruvate dioxygenase | 60000 |
| 969670 | 971625 | PA0887; acsA | Acetyl-coenzyme A synthetase | 33772 |
| 986818 | 989442 | PA0903; alaS | Alanyl-tRNA synthetase | 45081 |
| **1041689** | **1043404** | **PA0956; proS** | **Prolyl-tRNA synthetase** | **32904** |
| **1043983** | **1045314** | **PA0958; oprD** | **Basic amino acid, basic peptide and imipenem outer membrane porin** | **101706** |
| **1048459** | **1050234** | **PA0963; aspS** | **Aspartyl-tRNA synthetase** | **60414** |
| 1050301 | 1051047 | PA0964; pmpR | PqsR-mediated PQS regulator | 26820 |
| 1053848 | 1054543 | PA0969; tolQ | Tolq protein | 25115 |
| **1056049** | **1057347** | **PA0972; tolB** | **Tolb protein** | **33250** |
| **1057400** | **1057906** | **PA0973 ; oprL** | **Peptidoglycan associated lipoprotein** | **29407** |
| 1161856 | 1162977 | PA1074; braC | Branched-chain amino acid transport protein | 48761 |
| **1183058** | **1184524** | **PA1092; fliC** | **Flagellin type B** | **148757** |
| **1185060** | **1186484** | **PA1094; fliD** | **Flagellar capping protein** | **42861** |
| 1249907 | 1251154 | PA1155; nrdB | Tyrosyl radical-harboring component of class Ia ribonucleotide reductase | 37314 |
| **1251418** | **1254309** | **PA1156; nrdA** | **Catalytic component of class Ia ribonucleotide reductase** | **114872** |
| 1277006 | 1277608 | PA1178; oprH | PhoP/Q & low Mg2+ inducible outer membrane protein H1 | 62432 |
| 1277688 | 1278365 | PA1179; phoP | Two-component response regulator | 32271 |
| 1399231 | 1400505 | PA1288; | Probable outer membrane protein | 69445 |
| 1455815 | 1456723 | PA1342; | Probable binding protein component of ABC transporter | 32104 |
| **1689557** | **1690513** | **PA1552; ccoP1** | **Cytochrome c oxidase, cbb3-type, ccop subunit** | **51321** |
| **1690701** | **1691312** | **PA1553; ccoO1** | **Cytochrome c oxidase, cbb3-type, ccoo subunit** | **49955** |
| **1691327** | **1692754** | **PA1554; ccoN1** | **Cytochrome c oxidase, cbb3-type, ccon subunit** | **107679** |
| 1719109 | 1720395 | PA1580; gltA | Citrate synthase | 45462 |
| **1720744** | **1721130** | **PA1581; sdhC** | **Succinate dehydrogenase (C subunit)** | **33505** |
| 1721124 | 1721492 | PA1582; sdhD | Succinate dehydrogenase (D subunit) | 43170 |
| **1721496** | **1723268** | **PA1583; sdhA** | **Succinate dehydrogenase (A subunit)** | **166232** |
| **1723280** | **1723987** | **PA1584; sdhB** | **Succinate dehydrogenase (B subunit)** | **61151** |
| **1724244** | **1727075** | **PA1585; sucA** | **2-oxoglutarate dehydrogenase (E1 subunit)** | **268198** |
| 1727118 | 1728347 | PA1586; sucB | Dihydrolipoamide succinyltransferase (E2 subunit) | 87469 |
| 1728416 | 1729852 | PA1587; lpdG | Lipoamide dehydrogenase-glc | 120185 |
| **1730181** | **1731347** | **PA1588; sucC** | **Succinyl-coa synthetase beta chain** | **188255** |
| 1731347 | 1732234 | PA1589; sucD | Succinyl-coa synthetase alpha chain | 77181 |
| 1737536 | 1739440 | PA1596; htpG | Heat shock protein | 53309 |
| 1751674 | 1752891 | PA1609; fabB | Beta-ketoacyl-ACP synthase I | 46725 |
| **1752903** | **1753418** | **PA1610; fabA** | **Beta-hydroxydecanoyl-ACP dehydrase** | **29832** |
| **1910685** | **1912211** | **PA1767;** | **Hypothetical protein** | **27476** |
| **1914037** | **1916412** | **PA1770; ppsA** | **Phosphoenolpyruvate synthase** | **52821** |
| **1920568** | **1921065** | **PA1776; sigX** | **ECF sigma factor** | **25288** |
| **1921174** | **1922226** | **PA1777; oprF** | **Major porin and structural outer membrane porin** | **170951** |
| **1935035** | **1937644** | **PA1787; acnB** | **Aconitate hydratase 2** | **140045** |
| 1943067 | 1944737 | PA1794; glnS | Glutaminyl-tRNA synthetase | 42729 |
| 1944747 | 1946129 | PA1795; cysS | Cysteinyl-tRNA synthetase | 29603 |
| **1952665** | **1953975** | **PA1800; tig** | **Trigger factor** | **110351** |
| **1954069** | **1954710** | **PA1801; clpP** | **ClpP ATP-dependent protease** | **36898** |
| 1954815 | 1956095 | PA1802; clpX | Clpx; ATP-dependent protease | 73668 |
| 1956227 | 1958623 | PA1803; lon | Lon protease; ATP-dependent protease | 40588 |
| 1959263 | 1961128 | PA1805; ppiD | Peptidyl-prolyl cis-trans isomerase D | 43646 |
| 2458295 | 2460283 | PA2235; pslE | Protein involved in exopolysaccharide biosynthesis | 25915 |
| 2479299 | 2480744 | PA2252; | Probable AGCS sodium/alanine/glycine symporter | 37425 |
| 2655187 | 2657634 | PA2398; fpvA | Ferripyoverdine receptor | 40997 |
| 2743800 | 2746679 | PA2445; gcvP2 | Glycine cleavage system protein P2 | 53660 |
| **2761921** | **2778804** | **PA2462;** | **Hypothetical protein** | **48020** |
| 2965546 | 2966802 | PA2623; icd | Isocitrate dehydrogenase | 42767 |
| **2967161** | **2969386** | **PA2624; idh** | **Isocitrate dehydrogenase** | **60605** |
| 2983963 | 2985744 | PA2639; nuoD | NADH dehydrogenase I chain C,D | 37038 |
| 2986243 | 2987589 | PA2641; nuoF | NADH dehydrogenase I chain F | 23942 |
| 2987721 | 2990438 | PA2642; nuoG | NADH dehydrogenase I chain G | 65445 |
| **2990435** | **2991430** | **PA2643; nuoH** | **NADH dehydrogenase I chain H** | **23219** |
| 2992853 | 2994700 | PA2647; nuoL | NADH dehydrogenase I chain L | 29317 |
| 2994728 | 2996257 | PA2648; nuoM | NADH dehydrogenase I chain M | 24951 |
| 3096130 | 3098508 | PA2735; | Probable restriction-modification system protein | 25154 |
| **3100116** | **3102494** | **PA2739; phe** | **T ,phenylalanyl-tRNA synthetase, beta subunit** | **35631** |
| 3102529 | 3103545 | PA2740; pheS | Phenylalanyl-tRNA synthetase, alpha-subunit | 24447 |
| 3103643 | 3103999 | PA2741; rplT | 50S ribosomal protein L20 | 32283 |
| 3104023 | 3104217 | PA2742; rpmI | 50S ribosomal protein L35 | 36311 |
| **3104279** | **3104830** | **PA2743; infC** | **Translation initiation factor IF-3** | **50663** |
| **3104830** | **3106752** | **PA2744; thrS** | **Threonyl-tRNA synthetase** | **35138** |
| 3120073 | 3121350 | PA2760; oprQ | Outer membrane porin, OprD family | 58485 |
| 3204514 | 3205080 | PA2851; efp | Translation elongation factor P | 23161 |
| 3310792 | 3311721 | PA2951; etfA | Electron transfer flavoprotein alpha-subunit | 28302 |
| **3311721** | **3312470** | **PA2952; etfB** | **Electron transfer flavoprotein beta-subunit** | **39988** |
| 3323574 | 3324818 | PA2965; fabF1 | Beta-ketoacyl-acyl carrier protein synthase II | 28730 |
| 3325379 | 3326122 | PA2967; fabG | 3-oxoacyl-[acyl-carrier-protein] reductase | 32594 |
| **3332881** | **3336054** | **PA2976; rne** | **Ribonuclease E** | **54046** |
| 3359269 | 3360654 | PA3001; | Probable glyceraldehyde-3-phosphate dehydrogenase | 46597 |
| 3436578 | 3441440 | PA3068; gdhB | NAD-dependent glutamate dehydrogenase | 76963 |
| 3495446 | 3498205 | PA3115; fimV | Motility protein fimv | 27368 |
| **3543764** | **3545074** | **PA3159; wbpA** | **UDP-N-acetyl-d-glucosamine 6-Dehydrogenase** | **36089** |
| **3548110** | **3549789** | **PA3162; rpsA** | **30S ribosomal protein S1"** | **211515** |
| 3556427 | 3559198 | PA3168; gyrA | DNA gyrase subunit A | 43769 |
| 3982021 | 3984009 | PA3554; arnA | Methionyl-tRNA formyltransferase | 23778 |
| 4068677 | 4069966 | PA3635; eno | Enolase | 28672 |
| 4070012 | 4070857 | PA3636; kdsA | 2-dehydro-3-deoxyphosphooctonate aldolase | 27945 |
| **4070860** | **4072488** | **PA3637; pyrG** | **CTP synthase** | **51415** |
| 4078808 | 4080223 | PA3641; | Probable amino acid permease | 23699 |
| **4085062** | **4087455** | **PA3648; opr86** | **Outer membrane protein** | **31215** |
| 4093167 | 4094036 | PA3655; tsf | Elongation factor Ts | 66435 |
| 4094167 | 4094907 | PA3656; rpsB | 30S ribosomal protein S2 | 81829 |
| 4140884 | 4142389 | PA3700; lysS | Lysyl-tRNA synthetase | 27787 |
| **4195400** | **4196158** | **PA3743; trmD** | **tRNA (guanine-N1)-methyltransferase** | **48586** |
| 4196165 | 4196692 | PA3744; rimM | 16S rRNA processing protein | 39921 |
| 4215544 | 4219440 | PA3763; purL | Phosphoribosylformylglycinamidine synthase | 29697 |
| 4225660 | 4227237 | PA3769; guaA | GMP synthase | 24641 |
| 4265306 | 4266445 | PA3806; | Conserved hypothetical protein | 27352 |
| **4266470** | **4266901** | **PA3807; ndk** | **Nucleoside diphosphate kinase** | **24421** |
| **4270921** | **4272135** | **PA3814; iscS** | **L-cysteine desulfurase (pyridoxal phosphate-dependent)** | **30447** |
| 4277085 | 4278947 | PA3821; secD | Secretion protein | 33374 |
| 4291356 | 4294208 | PA3834; valS | Valyl-tRNA synthetase | 31121 |
| **4466925** | **4469546** | **PA3987; leuS** | **Leucyl-tRNA synthetase** | **30153** |
| 4622812 | 4624239 | PA4133; | Cytochrome c oxidase subunit (cbb3-type) | 28247 |
| **4753990** | **4754379** | **PA4237; rplQ** | **50S ribosomal protein L17** | **27386** |
| **4754423** | **4755424** | **PA4238; rpoA** | **DNA-directed RNA polymerase alpha chain** | **159089** |
| **4755447** | **4756067** | **PA4239; rpsD** | **30S ribosomal protein S4** | **100676** |
| **4756084** | **4756473** | **PA4240; rpsK** | **30S ribosomal protein S11** | **63819** |
| **4756492** | **4756848** | **PA4241;** | **Rpsm ,30S ribosomal protein S13** | **67720** |
| **4756979** | **4757095** | **PA4242; rpmJ** | **50S ribosomal protein L36** | **26311** |
| **4757124** | **4758452** | **PA4243; secY** | **Secretion protein secy** | **181289** |
| **4758453** | **4758887** | **PA4244; rplO** | **50S ribosomal protein L15** | **69931** |
| 4758891 | 4759067 | PA4245; rpmD | 50S ribosomal protein L30 | 36607 |
| **4759070** | **4759570** | **PA4246; rpsE** | **30S ribosomal protein S5** | **88042** |
| **4759574** | **4759924** | **PA4247; rplR** | **50S ribosomal protein L18** | **63135** |
| **4759935** | **4760468** | **PA4248; rplF** | **50S ribosomal protein L6** | **104015** |
| 4760480 | 4760872 | PA4249; rpsH | 30S ribosomal protein S8 | 67409 |
| 4761062 | 4761367 | PA4250; rpsN | 30S ribosomal protein S14 | 29254 |
| **4761381** | **4761920** | **PA4251; rplE** | **50S ribosomal protein L5** | **89675** |
| 4761940 | 4762254 | PA4252; rplX | 50S ribosomal protein L24 | 64997 |
| **4762267** | **4762635** | **PA4253; rplN** | **50S ribosomal protein L14** | **71554** |
| **4762659** | **4762925** | **PA4254; rpsQ** | **30S ribosomal protein S17** | **56218** |
| 4762928 | 4763119 | PA4255; rpmC | 50S ribosomal protein L29 | 42776 |
| **4763119** | **4763532** | **PA4256 ; rplP** | **50S ribosomal protein L16** | **80860** |
| **4763544** | **4764230** | **PA4257; rpsC** | **30S ribosomal protein S3** | **139761** |
| **4764243** | **4764575** | **PA4258; rplV** | **50S ribosomal protein L22** | **69723** |
| **4764588** | **4764863** | **PA4259; rpsS** | **30S ribosomal protein S19** | **59109** |
| **4764880** | **4765701** | **PA4260; rplB** | **50S ribosomal protein L2** | **164247** |
| 4765713 | 4766012 | PA4261; rplW | 50S ribosomal protein L23 | 69448 |
| 4766009 | 4766611 | PA4262; rplD | 50S ribosomal protein L4 | 122691 |
| **4766625** | **4767260** | **PA4263; rplC** | **50S ribosomal protein L3** | **128627** |
| **4767343** | **4767654** | **PA4264; rpsJ** | **30S ribosomal protein S10** | **46716** |
| **4767811** | **4769004** | **PA4265; tufA** | **Elongation factor Tu** | **151561** |
| **4769035** | **4771155** | **PA4266; fusA1** | **Elongation factor G** | **374495** |
| **4771186** | **4771656** | **PA4267; rpsG** | **30S ribosomal protein S7** | **101468** |
| **4771756** | **4772127** | **PA4268; rpsL** | **30S ribosomal protein S12** | **66741** |
| **4772279** | **4776478** | **PA4269; rpoC** | **DNA-directed RNA polymerase beta* chain** | **262206** |
| **4776544** | **4780617** | **PA4270; rpoB** | **DNA-directed RNA polymerase beta chain** | **254985** |
| **4780839** | **4781207** | **PA4271; rplL** | **50S ribosomal protein L7 / L12** | **50044** |
| **4781286** | **4781786** | **PA4272; rplJ** | **50S ribosomal protein L10** | **105224** |
| **4781985** | **4782680** | **PA4273; rplA** | **50S ribosomal protein L1** | **117208** |
| **4782680** | **4783111** | **PA4274; rplK** | **50S ribosomal protein L11** | **64755** |
| **4784316** | **4785509** | **PA4277; tufB** | **Elongation factor Tu** | **134770** |
| 4861653 | 4863176 | PA4333; | Probable fumarase | 23494 |
| 4893697 | 4894278 | PA4366; sodB | Superoxide dismutase | 27260 |
| 4898193 | 4899533 | PA4370; icmP | Insulin-cleaving metalloproteinase outer membrane protein | 43927 |
| 4915481 | 4917124 | PA4385; groEL | Major heat shock chaperone | 155497 |
| **4917175** | **4917468** | **PA4386; groES** | **Groes protein** | **34573** |
| 4933866 | 4936616 | PA4403; secA | Secretion protein | 47461 |
| **4938276** | **4939187** | **PA4406; lpxC** | **UDP-3-O-acyl-N-acetylglucosamine deacetylase** | **22923** |
| 4939300 | 4940484 | PA4407; ftsZ | Cell division protein | 42820 |
| 4962273 | 4963055 | PA4429; | Probable cytochrome c1 | 29231 |
| **4963055** | **4964266** | **PA4430** | **Probable cytochrome b** | **95378** |
| **4964266** | **4964859** | **PA4431;** | **Probable iron-sulfur protein** | **39359** |
| **4965109** | **4965501** | **PA4432; rpsI** | **30S ribosomal protein S9** | **25278** |
| **4965516** | **4965944** | **PA4433; rplM** | **50S ribosomal protein L13** | **51510** |
| 4984205 | 4985470 | PA4450; murA | UDP-N-acetylglucosamine 1-carboxyvinyltransferase | 30042 |
| 5013974 | 5015428 | PA4483; gatA | Glu-tRNA(Gln) amidotransferase subunit A | 30166 |
| 5015535 | 5016980 | PA4484; gatB | Glu-tRNA(Gln) amidotransferase subunit B | 22670 |
| **5037074** | **5038675** | **PA4500;** | **Probable binding protein component of ABC transporter** | **70263** |
| 5040405 | 5042000 | PA4502; | Probable binding protein component of ABC transporter | 37151 |
| 5042068 | 5043078 | PA4503; | Probable permease of ABC transporter | 29148 |
| 5043090 | 5044001 | PA4504; | Probable permease of ABC transporter | 23516 |
| 5044054 | 5045028 | PA4505; | Probable ATP-binding component of ABC transporter | 33663 |
| **5106951** | **5109782** | **PA4560; ileS** | **Isoleucyl-tRNA synthetase** | **46775** |
| 5116313 | 5116624 | PA4568; rplU | 50S ribosomal protein L21 | 37126 |
| 5146907 | 5148571 | PA4595; | Probable ATP-binding component of ABC transporter | 22866 |
| 5160738 | 5161991 | PA4602; glyA3 | Serine hydroxymethyltransferase | 28390 |
| 5163788 | 5165854 | PA4606; | Conserved hypothetical protein | 25570 |
| 5208464 | 5209987 | PA4640; mqoB | Malate:quinone oxidoreductase | 24503 |
| **5238408** | **5239349** | **PA4670; prs** | **Ribose-phosphate pyrophosphokinase** | **37520** |
| **5239466** | **5240080** | **PA4671;** | **Probable ribosomal protein L25** | **49381** |
| 5243178 | 5245406 | PA4675; | Probable tonb-dependent receptor | 22828 |
| 5272455 | 5273471 | PA4694; ilvC | Ketol-acid reductoisomerase | 24836 |
| 5274008 | 5275732 | PA4696; ilvI | Acetolactate synthase large subunit | 26641 |
| **5323374** | **5325479** | **PA4740; pnp** | **Polyribonucleotide nucleotidyltransferase** | **66150** |
| **5327427** | **5329949** | **PA4744; infB** | **Translation initiation factor IF-2** | **78475** |
| **5329977** | **5331458** | **PA4745; nusA** | **N utilization substance protein A** | **52263** |
| 5335782 | 5337701 | PA4751; ftsH | Cell division protein ftsh | 26703 |
| 5339864 | 5343085 | PA4756; carB | Carbamoylphosphate synthetase large subunit | 80695 |
| 5343767 | 5344903 | PA4758; carA | Carbamoyl-phosphate synthase small chain | 29997 |
| **5347198** | **5349111** | **PA4761; dnaK** | **DnaK protein** | **89547** |
| 5362146 | 5363195 | PA4774; | Hypothetical protein | 26273 |
| 5398627 | 5401707 | PA4812; fdnG | Formate dehydrogenase-O, major subunit | 26822 |
| 5442792 | 5444141 | PA4848; accC | Biotin carboxylase | 54367 |
| 5449046 | 5450653 | PA4854; purH | Phosphoribosylaminoimidazolecarboxamide formyltransferase | 23245 |
| 5535685 | 5536131 | PA4932; rplI | 50S ribosomal protein L9 | 31799 |
| 5536153 | 5537022 | PA4933; | Hypothetical protein | 84983 |
| 5537059 | 5537289 | PA4934; rpsR | 30S ribosomal protein S18 | 39171 |
| **5537319** | **5537738** | **PA4935; rpsF** | **30S ribosomal protein S6** | **60208** |
| **5542073** | **5543365** | **PA4938; purA** | **Adenylosuccinate synthetase** | **32662** |
| **5545786** | **5546988** | **PA4942; hflK** | **Protease subunit** | **37504** |
| 5547083 | 5548384 | PA4943; | Probable GTP-binding protein | 36902 |
| **5623040** | **5624797** | **PA5005;** | **Probable carbamoyl transferase** | **25074** |
| 5636156 | 5638804 | PA5015; aceE | Pyruvate dehydrogenase | 106941 |
| 5638949 | 5640592 | PA5016; aceF | Dihydrolipoamide acetyltransferase | 30186 |
| **5675714** | **5677858** | **PA5040; pilQ** | **Type 4 fimbrial biogenesis outer membrane protein** | **38404** |
| 5683471 | 5684739 | PA5046; | Malic enzyme | 31778 |
| 5716848 | 5718425 | PA5078; | Conserved hypothetical protein | 24478 |
| **5762659** | **5764476** | **PA5117; typA** | **Regulatory protein** | **37500** |
| **5766484** | **5767893** | **PA5119; glnA** | **Glutamine synthetase** | **35974** |
| 5826135 | 5828039 | PA5174; | Probable beta-ketoacyl synthase | 25623 |
| **5844468** | **5846009** | **PA5192; pckA** | **Phosphoenolpyruvate carboxykinase** | **55307** |
| **5853585** | **5855924** | **PA5201;** | **Conserved hypothetical protein** | **28722** |
| 5902386 | 5904596 | PA5242; ppk | Polyphosphate kinase | 24553 |
| 5972178 | 5973476 | PA5304; dadA | D-amino acid dehydrogenase, small subunit | 37600 |
| 5982097 | 5983590 | PA5312; | Probable aldehyde dehydrogenase | 23900 |
| **6006231** | **6008336** | **PA5338; spoT** | **Guanosne-3',5'-bis(diphosphate) 3'-pyrophosphohydrolase** | **25367** |
| **6109260** | **6110684** | **PA5429; aspA** | **Aspartate ammonia-lyase** | **25871** |
| **6248278** | **6249654** | **PA5554; atpD** | **ATP synthase beta chain** | **163874** |
| **6249685** | **6250545** | **PA5555; atpG** | **ATP sythase gamma chain** | **124009** |
| **6250596** | **6252140** | **PA5556; atpA** | **ATP sythase alpha chain** | **225414** |
| 6252159 | 6252695 | PA5557; atpH | ATP synthase delta chain | 93315 |
| **6252707** | **6253177** | **PA5558; atpF** | **ATP sythase B chain** | **84252** |
| **6253235** | **6253492** | **PA5559; atpE** | **Atp synhase C chain** | **41655** |
| **6253542** | **6254411** | **PA5560; atpB** | **ATP sythase A chain** | **54254** |
| **6261828** | **6263564** | **PA5568;** | **Consered hypothetical protein** | **40224** |

**Table S3: Sequence motifs associated with cleavage sites.** pRNA-Seq peaks were aligned and examined for potential patterns of nuclease digestion in a 10-nt window upstream and downstream of the cleavage site with the highest coverage in the window (dominant site). A single (global) motif was predominant within the data. K-mean clustering decomposed the RNA digestion patterns into five distinct classes that correlated with specific RNA motifs. Nucleotides present at a given distance from the cleavage site in 10-30% of the sequences are depicted in lowercase letters. Nucleotides present at in 31-64% of the sequences are depicted in uppercase letters and nucleotides present in 65% or more of the sequences are depicted in bold uppercase letters. The cleavage site is indicated by a ↓ symbol.

| **Peak Shape** | **Sequence Motif** |
| --- | --- |
| Global | [(A,C,g,u)(A,C,g,u)(G,a,c)(A,g,u) ↓ (A,c,u)(**C**,u)(A,c,g)(C,a,g,u)(C,a,g)] |
| Sharp | [(A,C,g,u)(A,C,u)(**G**,a,c)(A,g,u) ↓ (**A**,c,g)(**C**,u)(**A**,c,g)(C,a,g,u)(C,a,g)] |
| Tail L | [(A,C)(A,c,g,u)(**G**,a,u)(A,g,u) ↓ (C,U,a)(C,u)(A,c,g)(A,C,g,u)(C,a,g)] |
| Tail R | [(A,C,u)(A,C,u)(G,a,c,u)(A,G) ↓ (A,c,g,u)(**U**,c)(C,a,g,u)(A,C,g)(C,a,g)] |
| Twin L | [(A,c,g,u)(A,C,u)(G,a,c,u)(G,a,c,u) ↓ (A,c,u)(C,U)(A,G,c)(a,c,g,u)(G,a,c,u)] |
| Twin R | [(A,c,g,u)(C,a,g,u)(G,a,c,u)(A,c,g,u) ↓ (A,c,u)(C,U)(**A**)(C,U,a)(A,c,g)] |

**Table S4: p-values for promoters matching motif associated with virulence factors.** Pairwise distances were calculated for all PAO1 promoters. The promoter of a virulence factor of interest (PA1452, *flhA*) and the 10 most similar promoters to the virulence factor promoter were then used for motif prediction using MEME v4.8.1 [68] in an effort to identify novel members of regulons. The following is a list of the genes downstream of the promoter motif. Of the 10 promoters recovered in the search, three were upstream of other virulence factors and three were upstream of hypothetical proteins, which could play un-described roles in the virulence process.

| **Locus** | **Gene Name** | **Gene Product** | **Function** | **P-value** |
| --- | --- | --- | --- | --- |
| PA1452 | *flhA* | Flagellar biosynthesis protein | Adherence, Flagella | 2.57e-12 |
| PA1098 | *fleS* | Two-component sensor | Adherence, Flagella | 1.25e-11 |
| PA1441 | *fliK* | Putative flagellar hook-length control protein flik | Adherence, Flagella | 1.46e-11 |
| PA1094 | *fliD* | Flagellar capping protein flid | Adherence, Flagella | 6.99e-12 |
| PA1342 |  | Probable binding protein component of ABC transporter | Transport of small molecules | 1.48e-10 |
| PA0357 | *mutM* | Formamidopyrimidine-DNA glycosylase | DNA replication, recombination, modification and repair | 5.79e-06 |
| PA4581.1 |  | tRNA-Arg | Non-coding RNA gene | 1.26e-13 |
| PA4436 |  | Probable transcriptional regulator | Transcriptional regulators | 8.71e-07 |
| PA4739 |  | Conserved hypothetical protein | Hypothetical, unclassified, unknown | 5.87e-12 |
| PA0574 |  | Hypothetical protein | Hypothetical, unclassified, unknown | 6.12e-07 |
| PA5506 |  | Hypothetical protein | Hypothetical, unclassified, unknown | 3.07e-06 |

**Table S5A**: **Summary of predicted RpoN binding sites and adjacent downstream genes in *Pseudomonas aeruginosa* PAO1**. A position specific weighted profile generated from known RpoN binding sites was used to search putative promoter regions identified upstream of predicted TSS. TSS predictions are based on identification of 5’ triphosphates matching criteria specified by our algorithm (see Methods).

| **Down-stream gene** | **Start-Stop** | **Predicted RpoN binding sequence**  **Sequence** | **Down-stream gene description** | **% Con-fidence** | **Evidence for regulation by RpoN (PMID) ^2^** |
| --- | --- | --- | --- | --- | --- |
| PA0246 | 279456-279469 | GGCACCTGTCCTAC | probable major facilitator superfamily (MFS) transporter | 87 |  |
| PA0529 | 587950-587963 | GGCACTAGTCTAGC | conserved hypothetical protein | 88 |  |
| PA0730 | 799129-799142 | GGCACGCCGGCTGC | probable transferase | 89 |  |
| PA1035 | 1123883-1123896 | AGCACAATCACTGC | hypothetical protein | 86 |  |
| PA1077 | 1164215-1164228 | GGCACGGGCCTTGC | flagellar basal-body rod protein FlgB | 98 | 14617143 |
| PA1082 | 1168213-1168226 | GGCGCGGGTTTTGC | flagellar basal-body rod protein FlgG | 91 | 4617143 |
| PA1094 | 1184994-1185007 | GGCATGGTGCTTGC | flagellar capping protein FliD | 92 | 9488388 |
| PA1098 | 1189104-1189117 | GGCACGGGTATTGC* | two-component sensor | 97 | 9287015 |
| PA1269 | 1379231-1379244 | GGCACGCGCCAAGC | probable transcriptional regulator | 85 |  |
| PA1342 | 1456854-1456867 | GGCATGCATCCTGC | probable binding protein component of ABC transporter | 86 |  |
| PA1390 | 1511060-1511073 | GGCCCATTTTCTGC | probable glycosyl transferase | 85 |  |
| PA1441 | 1570430-1570443 | GGCCCGGACCTTGC | putative flagellar hook-length control protein FliK | 91 |  |
| PA1452 | 1580221-1580234 | GGAACGGTTCCTGC* | flagellar biosynthesis protein FlhA | 91 | 14617143 |
| PA1571 | 1712813-1712826 | GGCACGCCGACTGC | hypothetical protein | 89 |  |
| PA1642 | 1787197-1787210 | GGCGCCGGCTTTGC | selenophosphate synthetase | 86 |  |
| PA1770 | 1913888-1913901 | GGAACGATTCTTGG | phosphoenolpyruvate synthase | 85 |  |
| PA1851 | 2010878-2010891 | GGCACGCCGCATGC | hypothetical protein | 90 |  |
| PA1915 | 2090196-2090209 | GGCGCGCTGCCTGC | hypothetical protein | 89 |  |
| PA2519 | 2837210-2837223 | GGCGGAGGGCTTGC | transcriptional regulator XylS | 88 |  |
| PA2654 | 3003326-3003339 | TGCACGGTTCTTGC | probable chemotaxis transducer | 93 |  |
| PA2664 | 3013820-3013833 | GGCACGCTTCCTGA* | flavohemoprotein | 87 | 15937158 |
| PA2665 | 3013851-3013864 | TTCACGGGCCTTGC | Transcriptional activator of flavohemoglobin, FhpR | 85 |  |
| PA2945 | 3305802-3305815 | GGCCCGCGCACTGC | conserved hypothetical protein | 86 |  |
| PA3476 | 3889873-3889886 | GGCAGGTTGCCTGC* | autoinducer synthesis protein RhlI | 88 | 12670680 |
| PA3959 | 4438561-4438574 | GGCGCGCCGCATGC | hypothetical protein | 85 |  |
| PA4022 | 4502190-4502203 | GGCCCGGCCCTTGC | probable aldehyde dehydrogenase | 90 |  |
| PA4496 | 5031406-5031419 | GGCACATGCCCTGC | probable binding protein component of ABC transporter | 93 |  |
| PA4581.1 | 5130922-5130935 | GGCACGCCGCTCGC | tRNA-Arg | 87 |  |
| PA4739 | 5323168-5323181 | GGCACAGCGCTTGC | conserved hypothetical protein | 96 |  |
| PA5063 | 5702608-5702621 | GGCACAGCACTTTC | ubiquinone biosynthesis methyltransferase UbiE | 89 |  |
| PA5262 | 5924385-5924398 | GCCACGGGCCTTGC | alginate biosynthesis protein AlgZ/FimS | 90 |  |
| PA5267 | 5933454-5933467 | GGCACGAAGCTTGT | secreted protein Hcp | 87 |  |

* Sequence was included in the alignment that generated the profile.

^1^ Percent confidence for the hit identified by sequence profile. Motifs with a threshold reporting percentage greater than or equal to 85% were included in this analysis.

**^2^** Indicates a reference describing evidence for regulation of the gene by RpoN.

**Table S5B:** **Function classes associated with genes downstream of predicted RpoN binding sites**. Classification is based on PseudoCAP function classes available at the *Pseudomonas* Genome Database (www.pseudomonas.com).

| **Function Class** | **Count** |
| --- | --- |
| Hypothetical, unclassified, unknown | 7 |
| Transport of small molecules | 6 |
| Motility & Attachment | 6 |
| Adaptation, Protection | 5 |
| Putative enzymes | 4 |
| Two-component regulatory systems | 3 |
| Energy metabolism | 3 |
| Secreted Factors (toxins, enzymes, alginate) | 3 |
| Transcriptional regulators | 3 |
| Chemotaxis | 3 |
| Membrane proteins | 3 |
| Cell wall / LPS / capsule | 3 |
| Biosynthesis of cofactors, prosthetic groups and carriers | 2 |
| Translation, post-translational modification, degradation | 2 |
| Non-coding RNA gene | 1 |
| Amino acid biosynthesis and metabolism | 1 |
| Carbon compound catabolism | 1 |
| Central intermediary metabolism | 1 |
| DNA replication, recombination, modification and repair | 1 |

**Table S6: Fold change in RNA-Seq read density throughout the PAO1 genome.** Fold change in read density for each RNA-Seq library is shown in 0.5 Mbp increments along the PAO1 genome. Fold change is relative to the region with the lowest coverage (2,000,002-2,500,001 bp), which is at the terminus of replication.

| **Genome Region (bp)** | **RNA-Seq Library** | | | |
| --- | --- | --- | --- | --- |
|  | **PA0001** | **PA0004** | **A06026** | **A03674** |
| 1-500,001 | 4.0 | 3.0 | 2.0 | 3.0 |
| 500,002-1,000,001 | 27.8 | 5.4 | 15.0 | 9.2 |
| 1,000,002-1,500,001 | 5.1 | 3.7 | 3.1 | 3.7 |
| 1,500,002-2,000,001 | 9.2 | 6.2 | 4.5 | 5.4 |
| 2,000,002-2,500,001 | 1.0 | 1.0 | 1.0 | 1.0 |
| 2,500,002-3,000,001 | 1.8 | 1.7 | 1.7 | 3.6 |
| 3,000,002-3,500,001 | 3.0 | 2.8 | 3.1 | 2.7 |
| 3,500,002-4,000,001 | 2.4 | 2.2 | 2.4 | 2.4 |
| 4,000,002-4,500,001 | 3.3 | 3.3 | 3.5 | 3.2 |
| 4,500,002-5,000,001 | 28.6 | 11.6 | 22.5 | 15.6 |
| 5,000,002-5,500,001 | 19.2 | 5.8 | 13.4 | 7.7 |
| 5,500,002-6,000,001 | 6.7 | 4.3 | 3.4 | 4.9 |
| 6,000,002-6,264,404 | 17.5 | 4.1 | 11.0 | 6.9 |

**Table S7**: **Sequences of the oligonucleotides used in the preparation of the pRNA-Seq library.** The following oligonucleotides were used as primers or adapters during the construction of the pRNA-Seq library. See materials and methods for a complete description of the steps in which they were used.

| **DNA/RNA Oligo Name / Description** | **Oligo Sequence** |
| --- | --- |
| 17.71 AppDNA | AppGAA GAG CCT ACG ACG A |
| 17.50 (RNA adaptor) | AUC GUA GGC ACC UGA AA |
| 16.16 (AppDNA Rev. complement) | TCG TCG TAG GCT CTT C |
| 17.53 (17.50 DNA complement) | ATC GTA GGGC ACC TGA AA |

**Table S8: Fitted slopes and intercepts for data from H=1..100XX. Fit to G(H) = H_0_X^S^.** Power law fits to the dRNA-Seq and pRNA-Seq libraries as a function of strand orientation. Fits are to G(H) = H_i_(H)^S^ over the interval 3 < H < 85.

|  | dRNA-Seq (+) | dRNA-Seq (-) | pRNA-Seq (+) | pRNA-Seq (-) |
| --- | --- | --- | --- | --- |
| H_i_ | 7.78 X 10^5^ | 7.26 X 10^5^ | 1.86 X 10^3^ | 3.61 X 10^4^ |
| S | -2.08 | -1.97 | -1.24 | -1.53 |
| R | 0.988 | 0.991 | 0.994 | 0.995 |

**Table S9:** **List of primers utilized for confirmation of sRNAs by RT-qPCR.**

| **68836-69271-F** | CGAGCCGATAGGCGTCC |
| --- | --- |
| **68836-69271-R** | TTGGCTGGCCGTTGCTA |
| **99801-100048-F** | TTGTCCGATCTGCGGTGTC |
| **99801-00048-R** | TGCCGCAAGGCTTGTCA |
| **143349-517-F** | CGTCGGGTTTCGGAAAAA |
| **143349-517-R** | CCTGATTAGTTCTTTGGCTGACTCA |
| **326875-7066-F** | CGCCAGAAAGGAAGCTGTAATAG |
| **326875-7066-R** | CTCCCGGCTGACGGG |
| **334491-686-F** | GGCCGTTTTCAGGGCAT |
| **334491-686-R** | CCTTCGACGCGAGGTTTTT |
| **354527-742-F** | CGGGCTTCGCAGTGGA |
| **354527-742-R** | TGCCTTCCGAATCAGGGA |
| **707395-707685-F** | GCATAATGCGCCCCTTTTT |
| **707395-707685-R** | AACTGAACGGCCGCCC |
| **720091-720345-F** | CGCTGCAACACCGCTG |
| **720091-720345-R** | AGAAAGCGCCGCCGTATTA |
| **798865-799255-F** | AAATAGAGAGCGTCCGAAATCCT |
| **798865-799255-R** | TTCCTGCCCGGCCAAT |
| **830970-831031-F** | CGGATGAGCTGCGGGC |
| **830970-831031-R** | CCAGCACTACATCGCCCC |
| **883307-883582-F** | TGGTATTGCGGGACGCC |
| **883307-883582-R** | ACTCTTCTGAAGCAATCCCCTG |
| **1045414-733-F** | TCTTGTTGAGGTCGCTTCTCAA |
| **1045414-733-R** | CGGAACATGACATTTTTATTACAAGG |
| **1182820-3057-F** | AAAGCTCCGCCGGGAA |
| **1182820-3057-R** | GCTCAGGTGCCCCAAGAAT |
| **1254432-698-F** | GACTGTGAGTGCCTCCCTGG |
| **1254432-698-R** | AGGTATTGTGTTCGACGGCAA |
| **2761459-704-F** | TGAACCACGTGAAGCGGATA |
| **2761459-704-R** | AGGGAGGCTCCGCGAG |
| **2761599-911-F** | ACAGAACTTCAAAAGCCAGACTTTC |
| **2761599-911-R** | GGGCGGCTAGAGTCTACGC |
| **2964898-5137-F** | CCCCTTCGTCCTCGTGC |
| **2964898-5137-R** | CCTAGCCAGATTCGACTAACATTCA |
| **2977373-611-F** | CCTCGGCCTCCACCGT |
| **2977373-7611-R** | TTCCAGTCGCAATCTCGTCA |
| **3312577-693-F** | CAATACGGCAAAAGGGTGGT |
| **3312577-693-R** | TGAATTCTTTGGAAGCCTGATAGA |
| **3545572-872-F** | CCGAGCTTCGAATACGGCT |
| **3545572-872-R** | TGTGCGAGAAGATGCCAAGT |
| **3697226-433-F** | ACCGCTCATGGCGGC |
| **3697226-433-R** | GCGCCTAATAGCCCTGGG |
| **3930282-639-F** | CGCGGAGAATTACCGAGGA |
| **3930282-639-R** | ACCGCGTGAAAACCGCT |
| **4012653-735-F** | AAACCGGAGGGTCGTTTTT |
| **4012653-735-R** | TTCACAAAGGAATGCTGTCAA |
| **4536541-848-F** | GGATCTTGCGGGCGC |
| **4536541-848-R** | TCCGGATAAAGAGAGAACGGG |
| **5080450-630-F** | TTCTCCGCCTTGAAACCG |
| **5080450-630-R** | GCAGGGAAAAGAAGCCGATA |
| **5207898-5208463-F** | ATTAGCGCTTGAAACAGCCC |
| **5207898-5208463-R** | AGGCTCTGGTCATGAGGTATCC |
| **5224568-5224795-F** | CGTTTTCGACTCAGCCAAGG |
| **5224568-5224795-R** | GCTGGCGCCGTTCACTAA |
| **5309047-5309325-F** | CGCCCGAGAGGTCCTGATA |
| **5309047-5309325-R** | GCGTTGCTCAAACAGGACG |
| **5718503-5718753-F** | AAAAGAATGCCTGTTTCCAGTCA |
| **5718503-5718753-R** | TGCCCCCTGGTCTTCCA |
| **5973539-5973681-F** | TAGGAAGAGGCAGGCAGAAA |
| **5973539-5973681-R** | CCCCTAATTGTCCGGTTTTT |
| **5986186-5986427-F** | ACCCGCTGCATCCCG |
| **5986186-5986427-R** | TTCTGATATAAAGCTGCGCTCTTTT |
| **rsmY-F** | CAGGAAGCGCCAAAGACAAT |
| **rsmY-R** | TCCGTGCTACGCCACCA |
| **prrF1-F** | TCGCGAGATCAGCCGG |
| **prrF1-R** | GCCTGATGAGGAGATAATCTGAAGA |
| **prrF2-F** | ACTGGTCGCGAGGCCA |
| **prrF2-R** | GCCTGATGAGGAGATAATCTGAAGA |

**Perl Script to Remove 5’ Ends of Reads from pRNA-Seq Library**

#!/usr/bin/perl -w

use strict;

use warnings;

my $oligo = "ATCGTAGGCACCTGAAA";

my @oligoArray = split //, $oligo;

my $oligoLength = length($oligo);

my $maxErrors = 3;

my $TRIMMED = "false";

my $lineType = 0; ## 0=identifier, 1=sequence, 2=+, 3=quality scores

OUTER: while (<>) {

chomp;

my $line = $_;

#if ( $line =~ m/^\@.+/ && $lineType != 0 ) {

# print "ERROR processing read. Read identifier not formatted properly\n";

# exit;

#}

if ( $line eq "+" && $lineType != 2 ) {

print "ERROR processing read. \$lineType=$lineType and \$line=$line\n";

exit;

}

if ( $lineType == 0 ) {

print $line. "\n";

$lineType++;

next OUTER;

}

if ( $lineType == 1 ) {

### This is the sequence read line

if ( findOligo($line) == 1 ) {

$TRIMMED = "true";

$line =~ s/^.{17}(.+)$/$1/gi;

}

print $line. "\n";

$lineType++;

next OUTER;

}

if ( $lineType == 2 ) {

print $line. "\n";

$lineType++;

next OUTER;

}

if ( $lineType == 3 ) {

## This is the quality score line

if ($TRIMMED eq "true") {

$line =~ s/^.{17}(.+)$/$1/gi;

$TRIMMED="false";

}

print $line. "\n";

$lineType=0;

next OUTER;

}

}

sub findOligo {

my ($read) = @_;

my $errors = 0;

my @seq = split( //, $read );

for ( my $i = 0 ; $i < $oligoLength ; $i++ ) {

$errors++ unless ( $seq[$i] eq $oligoArray[$i] );

return 0 if ( $errors >= $maxErrors );

}

return 1;

}
